# Exercise Performance Measurement with Smartphone Embedded Sensor for Well-Being Management

**DOI:** 10.3390/ijerph13101001

**Published:** 2016-10-11

**Authors:** Chung-Tse Liu, Chia-Tai Chan

**Affiliations:** Department of Boimedical Engineering, National Yang-Ming University, Taipei 11221, Taiwan; g39904001@ym.edu.tw

**Keywords:** physical activity, motion sensors, feedback, well-being management

## Abstract

Regular physical activity reduces the risk of many diseases and improves physical and mental health. However, physical inactivity is widespread globally. Improving physical activity levels is a global concern in well-being management. Exercise performance measurement systems have the potential to improve physical activity by providing feedback and motivation to users. We propose an exercise performance measurement system for well-being management that is based on the accumulated activity effective index (AAEI) and incorporates a smartphone-embedded sensor. The proposed system generates a numeric index that is based on users’ exercise performance: their level of physical activity and number of days spent exercising. The AAEI presents a clear number that can serve as a useful feedback and goal-setting tool. We implemented the exercise performance measurement system by using a smartphone and conducted experiments to assess the feasibility of the system and investigated the user experience. We recruited 17 participants for validating the feasibility of the measurement system and a total of 35 participants for investigating the user experience. The exercise performance measurement system showed an overall precision of 88% in activity level estimation. Users provided positive feedback about their experience with the exercise performance measurement system. The proposed system is feasible and has a positive effective on well-being management.

## 1. Introduction

Sufficient physical activity has substantial benefits for health. Regular physical activity such as fast walking, running, and cycling reduces the risk of coronary heart disease, type 2 diabetes, and depression, as well as facilitating weight control [1,2,3,4]. Moreover, physical activity improves mental health and reduces cognitive impairment. However, 31.1% of adults worldwide are physically inactive [1]. Increasing physical activity is a global health care concern.

Wearable health care sensors have potential to improve physical activity levels. An inexpensive, accurate, and stable device that can assess physical activity in real-world environments can facilitate the management of personal health. A variety of activity monitoring devices has been developed for health promotion. Several technologies are available for the measurement and assessment of physical activity, such as doubly labeled water (DLW), indirect calorimetry, pedometers, accelerometers, heart rate measuring devices, and global positing systems [3]. For example, walking is a health-boosting activity, and pedometers can assist in motivating physical activity and tracking progress. A pedometer can function as an activity sensor, which is used to monitor physical activity for the purpose of health promotion. The global positioning system can be used to measure activity by computing the distances and speeds of outdoor activities (e.g., walking and running). In general, sensors such as accelerometers have the features of low cost, suitability for personal recording, and easy to use. In recent years, researchers have found that the level of activity is essential and that the intensity of activity is crucial. Data analysis algorithms are combined with wearable sensors. Activity recognition and activity level estimation technologies are applied in personal well-being management to address physical inactivity problems by providing accurate information to users. 

The estimation of activity level and activity recognition by acceleration signal is determined using regression techniques [5]. A classifier can classify input data into a labeled category. There are various well-known classifiers that have been implemented in smartphones such as support vector machine, K-nearest neighbor, decision tree, and neural networks [6,7]. Although most published studies have good accuracy in activity recognition problems, these studies differ in the recognition of activity type and in the position, type, and number of sensors. It is hard to directly compare different methods in classification problems [8]. However, some studies showed that decision trees and neural networks may have better accuracy in recognition problems [7,8]. Comparing decision trees and neural networks, decision trees have easy coding, fast prediction, lower battery consumption, and interpretability. 

Several studies have implemented activity recognition or activity level estimation approaches by applying accelerometers [9,10,11,12,13,14,15,16,17,18,19,20]. For example, Kwapisz et al. identified activity types through smartphones carried in the users’ pockets [11]. Weiss et al. established a smartphone-based activity recognition system to monitor personal health [12]. Such studies are useful and have contributed to well-being management. However, most of such studies have focused on activity measurement for a single period and providing feedback on the basis of this measurement. Regular physical activity over longer periods has not been examined. The World Health Organization (WHO) defines sufficient physical activity as at least 600 MET-min/week, which equates to approximately 75 min of high-intensity activity, 150 min of moderate-intensity activity, or 600 min of mixed-intensity activity. Consequently, if devices cannot accumulate activity records for a week, to meet this recommendation, users must calculate their weekly activity levels. In addition, the main purpose of the measurement of physical activity is to provide feedback to the user, thereby increasing motivation and enabling targets to be set and aimed toward [3,21]. Complicated feedback systems are a potential barrier to the popularization of devices; simple indicators are more suitable for public consumption. Studies have suggested that goal-setting can increase self-regulatory behavior and increase physical activity [22]. Similarly, goals must be set clearly to facilitate understanding.

The accumulated activity effective index (AAEI) was proposed to analyze physical activity on the basis of physical activity levels and the number of days spent exercising [23]. The AAEI system entails feedback being inputted to a numeric index every day irrespective of whether the user is resting or exercising. The AAEI can be applied to set goals for increasing physical activity or for maintaining sufficient physical activity. Because the AAEI is based on the number of days spent exercising, users can read the index every day to inspect their physical activity. Therefore, we propose an exercise performance measurement system that is based on the AAEI for generating an index that includes the levels of physical activity and the number of days spent exercising. The proposed mechanism for well-being management was implemented using a smartphone because smartphones have a user-friendly interface and embedded motion sensors. Users can set goals, understand their physical activity levels, and be motivated.

## 2. Materials and Methods

### 2.1. System Architecture

**Figure 1:**
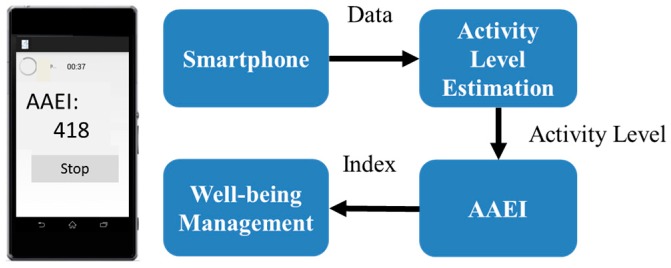
Function diagram of the exercise performance measurement system.

The exercise performance measurement system is implemented using a smartphone. For a motion sensor, the smartphone employs a triaxial accelerometer that is provided with a range of ±2 g. The sampling rate is 40 Hz. The smartphone is worn on the left upper arm by using phone accessories. A system function diagram is presented in Figure 1. The proposed mechanism includes an activity level estimation stage and an AAEI stage. The activity level estimation process transfers motion data to the activity level. The activity level estimation process comprises signal preprocessing, feature extraction, and an activity level estimation model. The AAEI stage calculates the index on the basis of the activity level and duration. When activated, the system can monitor running and walking. Users can read the AAEI after they have finished exercising.

### 2.2. Activity Level Estimation Mechanism

**Figure 2:**
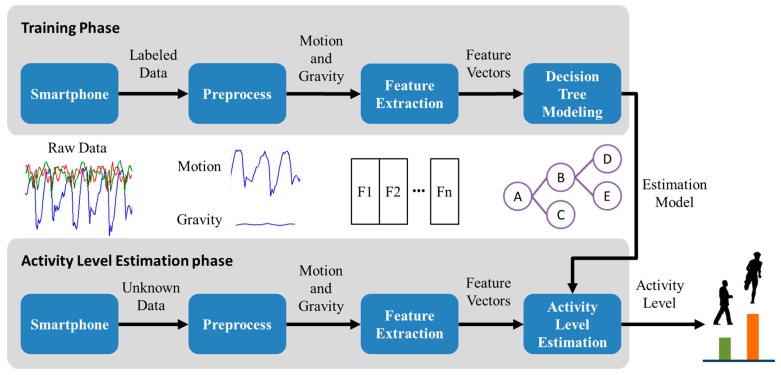
Framework of activity level estimation.

The activity level estimation comprises three components, namely signal preprocessing, feature extraction, and activity level estimation. Figure 2 illustrates the activity level estimation process. The training phase begins with the preprocessing of a time series data signal. The preprocessing function involves a low-pass filter used to separate gravity and motion data. The feature vector is then extracted from the motion data. The features are grouped into clusters according to the activity level and modeled through decision tree classification [8], a technique commonly used in data mining and extensively applied in many applications for classification that entails constrained requirements. When used for activity level estimation, decision trees are usually trained to learn a decision boundary between different activity level patterns. In the activity level estimation phase, the sampling data are processed through the same preprocessing and feature extraction functions as in the training phase. The activity level is outputted by the estimation model generated by the training phase. The details of the components are described in this subsection.

#### 2.2.1. Preprocessing

The acceleration signal is recorded by an accelerometer embedded in the smartphone and contains gravity and body movement acceleration. In the signal preprocessing, a low-pass filter with a cutoff frequency at 0.5 Hz is used to separate gravity and body movement signals [16]. The gravity component is obtained directly by applying the low-pass filter to the acceleration signal, whereas the body motion component is determined using the difference between the original signal and the gravity component. A window technique is used to divide the continuous body motion signal into segments. The time window size is defined as 2 s, because a period of 1–2 s provides a favorable trade-off between recognition speed and accuracy [15]. We observed that a larger window can smooth the signal features whereas a smaller window is sensitive to the signal features. The 2 s time window size of a body motion signal will be processed through feature extraction.

#### 2.2.2. Feature Extraction

Numerous features can be utilized to identify activity levels. Previous activity recognition studies extracted wide range of features to identify activity [6,8]. These features usually are time-domain and frequency-domain features. Time-domain features such as mean, variance, and maximum or minimum values, are generated directly from a time window. Frequency-domain features are applied to fast Fourier transform to generate frequency-based features. Frequency-domain features such as entropy, energy, and frequency can be used in activity recognition problems [5]. These features are used both in training and estimation phase. Smartphones have limited computational capacity. Therefore, to avoid excessive complexity, the number of features should be restricted. On the basis of previous studies [9,16,18], we selected 10 features: signal magnitude area (SMA, Equation (1)), signal magnitude vector (SMV, Equation (2)) [16], maximum y- and z-axis value of motion signal, and the first three magnitude values and frequencies of fast Fourier transformation. The extracted features form a feature vector per time segment. Each segment results in an activity level being produced according to the feature vector.

(1) $$\mathrm{SMA}=\frac{1}{t}\left(\int_{0}^{t}\left|x\left(t\right)\right|dt+\int_{0}^{t}\left|y\left(t\right)\right|dt+\int_{0}^{t}\left|z\left(t\right)\right|dt\right)$$

where *x*(*t*), *y*(*t*), and *z*(*t*) refer to the x-, y-, and z-axis samples, respectively.

(2) $$\mathrm{SMV}=\sqrt{{x_{i}}^{2}+{y_{i}}^{2}+{z_{i}}^{2}}$$

where $x_{i}$, $y_{i}$, and $z_{i}$ are the *i-*th sample of the x-, y-, and z-axis signal, respectively.

#### 2.2.3. Decision Tree Modeling

The activity categories considered in this study are outlined as follows: walking, fast walking, running, and stationary. The activity categories and corresponding activity levels are defined in Table 1. Researcher measures physical activity as energy expenditure using metabolic equivalent of task (MET) as unit to quantify activity level. One MET is defined as 1 kcal/kg/h. The MET value is independent of person and can be used to estimate intensity of physical activity. The intensity of sedentary activity is approximately 1 MET. Activity categories of light, moderate, and vigorous intensity are between 1 and 3, 3 and 6, and 6 and 9 METs, respectively. Activity of greater than 9 METs is extremely vigorous. 

Decision tree algorithm is an extensive machine learning method to solve classification problems of pattern recognition [8,15]. A decision tree is a hierarchical scheme that has a tree-like structure. A decision tree is composed of a set of interior nodes and terminal nodes. Each interior node of the decision tree is a threshold of one feature that makes a binary decision. The data is separated by interior node to a terminal node or next interior node. A terminal node represents one class of the classification problems. 

The training data through the preprocessing and feature extraction processes. The decision tree was produced by C4.5 and identifies various activity level categories. The participants were recruited to perform physical activity with a smartphone strapped on the left upper arm. In the training phase, the decision tree model with labeled feature vectors is used to construct an activity level estimation model. The activity level estimation model is validated by 10-fold cross validation. The generated activity level estimation model is then used in the recognition phase to estimate activity levels. The decision tree is a rule based algorithm that can be implemented in smartphone using *if-then* rules.

### 2.3. Accumulated Activity Effective Index

The AAEI is used to measure exercise performance after activity level estimation. The activity index calculator is a simple numeral indicator that determines the physical activity status of a user by estimating both the accumulated quantity of physical activity and the number of days spent exercising [23]. The index is estimated according to daily physical activity. The functions are described by Equations (3)–(5) and the parameters are shown in Table 2.

The term AAEI is an index of the accumulated physical activity in the time interval $\left(t_{1},t_{2}\right)$, where the base unit of time is the day. The design principles of the AAEI were: (1) AAEI increases with more physical activity, is steady in fixed physical activity, and decreases with less physical activity; (2) AAEI corresponds to days spent exercising; (3) AAEI decreases with resting days; (4) AAEI decreases more with continued resting; (5) AAEI decreases less at rest if user has exercised before; and (6) AAEI is at or near zero if the user does not exercise in seven days [23]. The AAEI, $I\left(t_{1},t_{2}\right)$, is estimated by previous AAEI ($I\left(t_{1},t_{2}-1\right)$), current quantity of physical activity ($k\left(M\right)\times MT\left(t_{2}\right)$), and a predictor ($E\left(t_{2}\right)$). The initial value of the AAEI evaluation process to be set was $I\left(t_{1},t_{2}-1\right)$ = 0, $E\left(t_{2}\right)$ = 0, and α was null. The quantity of physical activity (*MT*) is multiplication of activity level (*M*) and exercise duration (*T*). Activity level of light, moderate, vigorous, and extremely intense categories are 2, 4.5, 7.5, and 9 METs, respectively. The sedentary intensity does not accumulate into the AAEI. The parameter k(M)=0 when intensity is sedentary, otherwise k(M)=1. The predictor ($E\left(t_{2}\right)$) is a threshold that predicts the quantity of physical activity a user should take. Coefficient α is estimated based on accumulated quantity of physical activity and days spent exercising. $A\left(t_{1},t_{2}-1\right)$ is a value defined as $I\left(t_{1},t_{2}-1\right)$)/7 that represents the previous quantity of physical activity to estimate the predictor $E\left(t_{2}\right)$ and coefficient α.

(3) $$I\left(t_{1},t_{2}\right)=I\left(t_{1},t_{2}-1\right)+k\left(M\right)\times MT\left(t_{2}\right)-E\left(t_{2}\right)$$

(4) $$E\left(t_{2}\right)=A\left(t_{1},t_{2}-1\right)\times C^{-\alpha }$$

(5) $$\alpha =\overset{t_{2}-t_{1}}{\underset{i=1}{\sum }}\frac{MT\left(t_{2}-i\right)-A\left(t_{1},t_{2}-i\right)}{A\left(t_{1},t_{2}-i\right)}\times W^{\left(i-1\right)}$$

### 2.4. Prototype Implementation

**Figure 3:**
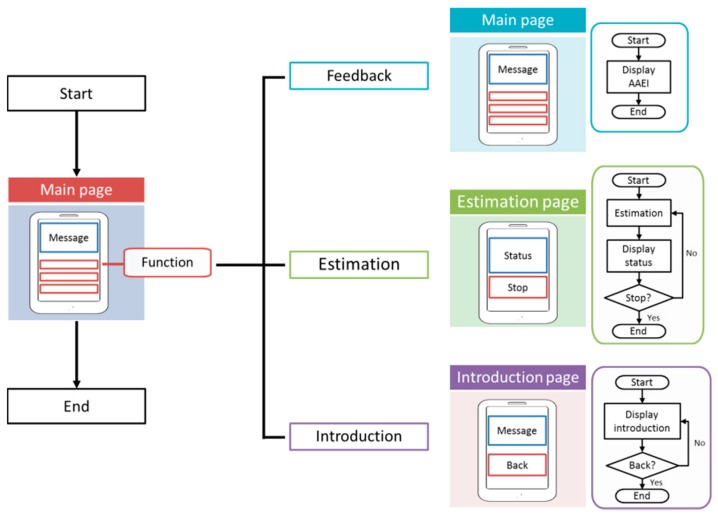
Exercise performance measurement system using Android for implementation.

The paper prototype system is implemented in Android system. The development of a paper prototype involves the following stepwise procedure: (1) Setting targets: The main target is the setting up of an exercise performance measurement and feedback system; (2) Setting up of the template and application workflow: The setting up of the initial template and application workflow; (3) Application function design: This entails creating the system context and constructing the prototype flowchart; (4) User interface design: Setting up a user-friendly interface after reconciling data gathered from the initial prototype; (5) Deployment and testing: Function reliability is tested step by step. Figure 3 shows the design and implementation of exercise performance measurement system. The application of the prototype system contains feedback, estimation, and introduction function. The feedback function can display AAEI value to user. The estimation function can estimate AAEI and the display status contains exercise duration, AAEI value, and quantity of physical activity. The estimation function requires preprocessing, an estimation model for activity level estimation, and feature extraction, as well as data access and a calendar for AAEI calculation. An introduction page for users is also necessary to the user. The introduction function displays an introduction of AAEI and a relation of physical activity and AAEI value.

### 2.5. Experiments

Experiments were conducted to examine the feasibility of the proposed system and investigate the user experience. The experiments had two parts: (1) verification of activity level estimation using a smartphone-embedded sensor; and (2) assessment of the user experience and feasibility of the exercise performance measurement system.

We recruited participants to investigate the feasibility of the activity level estimation in the first experiment. Each participant strapped a smartphone on their left upper arm. The participants were asked to walk and run on a treadmill. Walking and fast walking were performed at speeds of 1 to 7 km/h; each speed was maintained for at least 2 min at increments of 1 km/h. Running was performed at speeds of 5 to 9 km/h; each speed was performed for at least 2 min at increments of 1 km/h. All the participants were selected on the basis of their physical condition (such as not having heart disease or other bodily impairments). The participants could stop the experiment at any time if they did not wish to continue.

**Figure 4:**
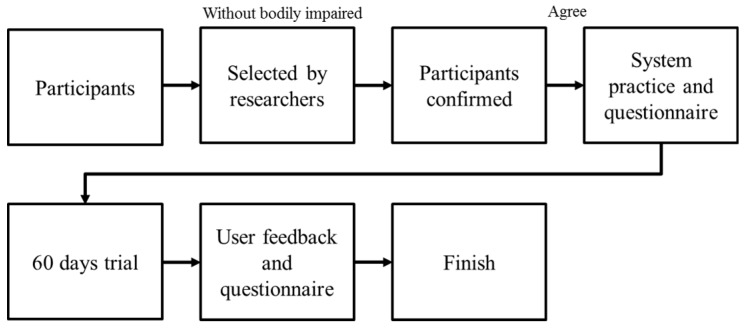
Flowchart of a real-world trial.

The second experiment was a real-world trial using the proposed exercise performance measurement. The participants were equipped with a smartphone for a short-term evaluation. We recruited two user groups. One group used the system for a single time trial. The single-time users tested the system for a few hours while walking or running, and subsequently completed a questionnaire. The other group underwent a 60-day trial. The experimental flowchart is shown in Figure 4. The participants were first selected by researchers to confirm that they were able to walk and run without any risk. After the participants agreed to the experimental process, the researchers demonstrated the system and let the participants learn to operate the system independently. The participants answered a physical activity questionnaire before and after the 60-day trial. At the end of the trial, the participants answered a questionnaire concerning the user experience and were asked to attend an interview with the researchers.

### 2.6. Ethical Statement

All subjects gave their informed consent for inclusion before they participated in the study. The study was conducted in accordance with the Declaration of Helsinki, and the protocol was approved by the Institutional Review Board of National Yang-Ming University (YM104065E). 

## 3. Results and Discussion

### 3.1. Activity Level Estimation

**Figure 5:**
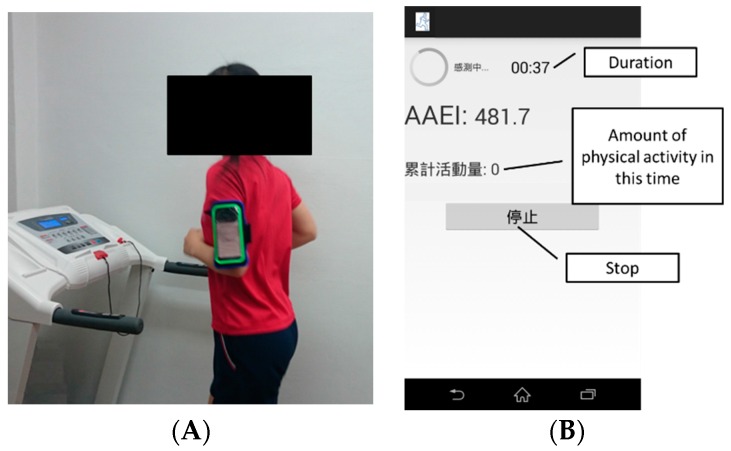
Data collection for activity level estimation. (**A**) Participant wearing smartphone on the left upper arm and performing exercise on a treadmill; (**B**) Smartphone screenshot during exercise measurement.

The experimental data were collected from 17 participants, comprising 11 males and 6 females. The age of the group was 22.5 ± 2.3 years (ranging from 20 to 30 years). Figure 5A shows a participant on a treadmill wearing a smartphone on the left upper arm. Figure 5B shows a screenshot of the smartphone during the exercise measurement. The data were collected for at least 2 min for each velocity measured. The participants were allowed to walk and run according to their own method.

**Figure 6:**
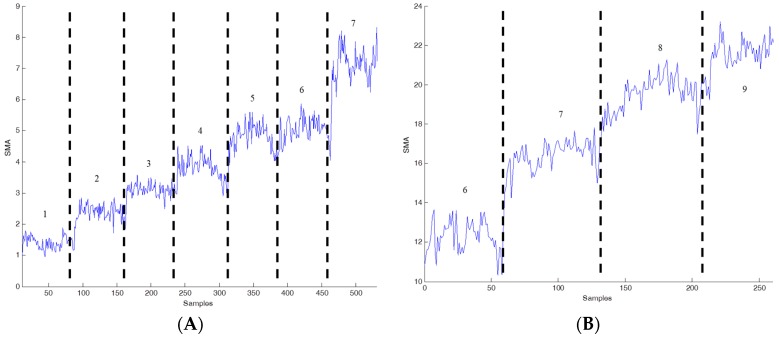
Different velocities with different SMA values. (**A**) SMA values during walking on the treadmill at velocities from 1 to 7 km/h; (**B**) SMA values during running on the treadmill at velocities from 6 to 9 km/h.

The raw accelerometer data were transformed into a set of features. The features were selected on the basis of previous effective performance [9,16,18]. These features are computationally simple and enabled activity levels to be estimated in near real time. Figure 6 shows SMA values for a range of walking and running velocities. Although the SMA values varied for different participants, the values tended to increase with the velocity. The SMA values derived for running were higher than those derived for walking. The SMA values are implemented as a measurement to calculate energy expenditure [16]. Therefore, the set of features including SMA value was applied for estimation using a decision tree model.

**Figure 7:**
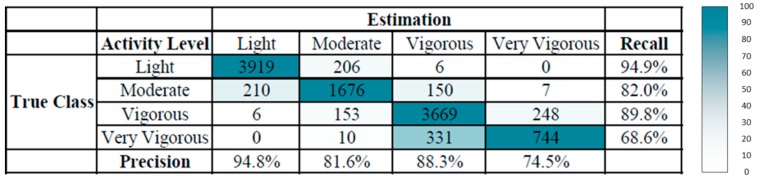
Confusion matrix of activity level estimation for walking, fast walking, and running tasks.

We applied all the intensity and activity types in the training phase. To demonstrate the performance of the activity level estimation, the precision and recall data are presented. Figure 7 shows the confusion matrix of the activity level estimation performed using the smartphone-embedded sensor during walking, fast walking, and running tasks on the treadmill. The overall precision of the activity level estimation was 88.2%. The precision level was highest for light-intensity physical activity. The precision levels for moderate and vigorous physical activity were lower than that for light activity. The extremely vigorous physical activity exhibited the lowest precision level, because of the large variance and overlap of the extracted features at vigorous activity levels. Moreover, the variation of features among participants was more pronounced when the velocity of the treadmill was higher. The training samples were possibly less compared with those collected in the other categories. The participants differed in cardiopulmonary function, and this affected their exercise performance at high velocity. According to the observation of this running task, wearing clothes and shoes and exercise habits may affect the activity level estimation. The data collected from the accelerometer for different activity categories and activity intensities may exhibit scattered variation because of user differences.

### 3.2. User Experience and System Performance

We recruited two groups to examine user experience. The first group contained single-time system users. Seventeen participants comprising 11 males and 6 females were recruited in the single-time user group. The average age of the single time user group was 22.5 ± 2.3 years (ranging from 20 to 30 years). They walked and ran for a specific period and then completed a questionnaire about the system. The second group contained participants subjected to a 60-day real-world trial experiment to assess the feasibility of the proposed mechanism. Eighteen participants (11 males and 7 females) with an average age of 27.1 ± 8.4 years (ranging from 22 to 60 years) were recruited to participate in the experiment. The participants were free to perform walking, fast walking, and running at any speed in any location. The real-world trial participants were asked to complete the questionnaire after the trial and meet with researchers.

**Figure 8:**
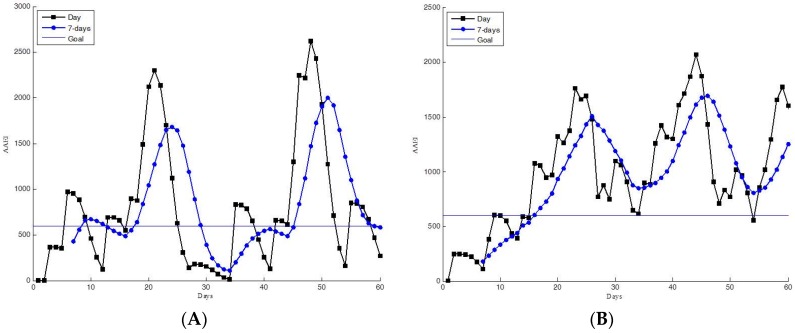
Participants with high AAEI values, as determined in the 60-day trial. (**A**,**B**) Several high AAEI values above 600.

**Figure 9:**
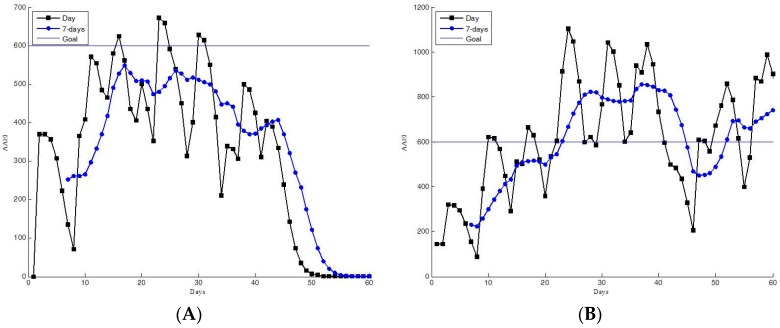
Participants with moderate AAEI values, as determined the 60-day trial. (**A**,**B**) Participants typically had AAEI values between 400 and 600.

**Figure 10:**
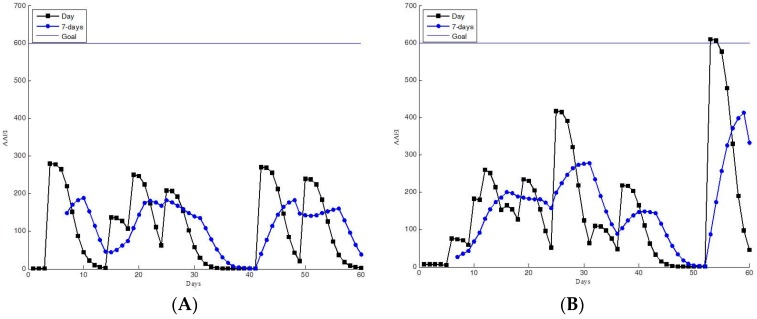
Participants with insufficient AAEI values, as determined in the 60-day trial. (**A**,**B**) AAEI values indicating insufficient activity but with occasional exercise.

**Figure 11:**
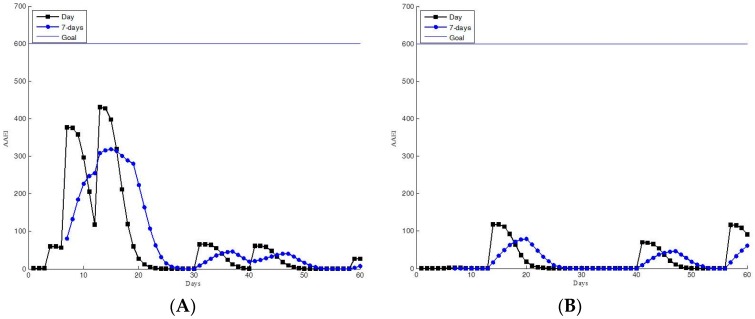
Participants with inactive AAEI values, as determined in the 60-day trial. (**A**,**B**) Low AAEI values indicating infrequent exercise.

The AAEI indicates the exercise performance of participants according to the accumulated amount of physical activity and number of days spent exercising. The participants can read their AAEI values to determine their personal exercise performance. The AAEI also indicates the trends in their exercise performance. Examples of estimated AAEIs from the 60-day trial group are shown in Figure 8, Figure 9, Figure 10 and Figure 11. The AAEI day line and seven-day average line are shown separately in the figures. We classified the exercise performance of the participants into four groups according to their AAEI values. We set 600 as the goal for all participants. The AAEI estimated for sufficient physical activity is 400–600 based on different exercising days and activity level. The AAEI takes days spent exercising into consideration. There will be a 30% decrease in a week three days of exercise are removed. There will be no decrease if user can take exercise every day [23]. We set 600 for the fitness goal because 600 is a high standard of sufficient physical activity. The four groups were high activity, moderate activity, insufficient activity, and inactive. The high- and moderate-activity groups exhibited sufficient physical activity, according to the WHO guidelines. The moderate-activity group had AAEI values between 400 and 600. Figure 8 shows the 60-day trial participants with high AAEI values. These participants scored AAEI values of more than 600. The participants typically had regular exercise habits and appeared to have training plans, and they occasionally accumulated extremely high levels of AAEI. Their environment and work schedule did not influence their exercise routines. Figure 9 shows the AAEI values derived for the participants in the moderate-activity group, indicating values between 400 and 600 (sometimes above 600, but not substantially). The participants in the moderate-activity group exercised, but less regularly than those in the high-activity group did. Their exercise performance appeared to be more affected by their environment and work schedule. Some days with bad weather (e.g., cold or rain) occurred during the 60-day trial, which may have reduced the participants’ motivation to engage in exercise. 

Figure 10 and Figure 11 show the data for the insufficient physical activity group. The participants in these groups did not have sufficient physical activity, according to the WHO guidelines. Figure 10 shows that the participants occasionally engaged in exercise. However, they seldom accumulated sufficient physical activity. This seems to be habitual behavior; another possible explanation is that the participants engaged in other types of exercise than walking and running. Figure 11 shows the data for the inactive participants. The low AAEI values indicate that the participants infrequently exercised. The exercise performance measurement system appeared not to motivate these participants sufficiently. 

Examining user experience is essential to evaluate the feasibility of the exercise performance measurement system. Users’ experiences were collected through the formal questionnaire presented in Table 3. Users could provide further feedback in comments after completing the formal questionnaire. Answers regarding the two groups’ experience of the exercise performance measurement system are shown in Table 3. The questions were answered with a 5-point scale (1 = *strongly disagree*; 2 = *disagree*; 3 = *normal*; 4 = *agree*; and 5 = *strongly agree*). In the single-time user group, the feedback was positive and all questions received responses above 4 points. The single-time user group agreed that the proposed AAEI was helpful for goal-setting and the management of well-being. The participants in this group typically assigned higher points than those in the 60-day trial group did. The user meetings and observations conducted by the researchers provided some explanations for this: First, the age ranges of the two groups differed. The single-time user group comprised younger participants than those in the 60-day trial group. The 60-day trial group contained a participant who was nearly 60 years of age. The elderly participants may have had more concerns about their health, but they did not exhibit curiosity about the system. Moreover, the novelty of the system may decrease with time. In the meeting with the 60-day trial group, some of the participants stated that the system must record more exercise types. Because the participants were not restricted to walking and running, they wanted to record and estimate more types of exercise and acquire feedback subsequently. The third explanation concerned convenience. Some participants stated that wearing the sensor or smartphone was inconvenient for them when exercising. They wanted to receive feedback after exercising but did not want to wear a device. Some participants reported forgetting to wear the device because of their unfamiliarity with it. Minimizing the device or incorporating the device into clothes may increase user satisfaction. In general, the two groups provided positive feedback about the exercise performance measurement system. Both groups agreed that the AAEI is clear and provides effective well-being management.

## 4. Conclusions

Regular physical activity improves physical and mental health. Increasing physical activity is essential for well-being management. We developed an exercise performance measurement system for well-being management that is based on the AAEI and incorporates a smartphone-embedded sensor. Users can set goals and assess their physical activity levels, as well as being motivated by the measurement system. The proposed exercise performance measurement system can generate a numeric index that is based on users’ exercise performance, namely their level of physical activity and number of days spent exercising. The index is a clear number that is useful for feedback and goal-setting. We implemented the exercise performance measurement system by using a smartphone and conducted experiments to validate the feasibility of the system and user experience. The exercise performance measurement system shows an overall precision of 88% in activity level estimation. Users provided positive feedback and agreed that the AAEI was clear. The AAEI can also be a tracking tool for examining the exercise history of users. The proposed exercise performance measurement system is feasible and has a positive effect on well-being management.

## Figures and Tables

**Table 1:** Activity category and corresponding activity level.

Activity	Activity Level
Stationary	Sedentary
Walking slowly (<2 km/h)	Light
Walking (2–4 km/h)	Light
Walking fast (5–7 km/h)	Moderate: 5–6 km/h
	Vigorous: 7 km/h
Running (6–9+ km/h)	Vigorous: 6–8 km/h
	Extremely vigorous: 9+ km/h

**Table 2:** AAEI parameters.

I: I≥0, an index	$\left(t_{1},t_{2}\right)$: time interval from $t_{1}$ to $t_{2}$, unit: day
k(M): $k\left(M\right)=\{\begin{array}{l} 0,\;M=sedentary\\ 1,\;otherwise \end{array}$	$MT\left(t_{2}\right)$: $MT\left(t_{2}\right)\geq 0$, activity level (M) multiple duration (T) in day $t_{2}$
$E\left(t_{2}\right)$: $E\left(t_{2}\right)\geq 0$	$A\left(t_{1},t_{2}-1\right)$: $I\left(t_{1},t_{2}-1\right)$)/7
C: C=2, a constant defined as 2	α: −2≤α<∞, a coefficient estimated by accumulated physical activity
i: *i*-th days before	W: W=0.5, a constant of attenuation defined as 0.5

**Table 3:** User experience of the exercise performance measurement system. User experience of the single-time user group and 60-day trial group. Scale ranged from 1 (*strongly disagree*) to 5 (*strongly agree*).

Questions	Single Time Used (*n* = 17)	60 Days Trial (*n* = 18)
Do you agree that the AAEI can help me for fast understand my exercise performance?	4.2	3.9
Do you agree that the AAEI can help me for goal-setting?	4.1	3.8
Do you agree that the AAEI is clear and definite?	4.2	4.1
Do you agree that the AAEI can help me to increase or maintain sufficient physical activity?	4.2	3.6
Do you agree that the AAEI can be shared with people with less privacy concern?	4.3	4
Overall, do you agree that the AAEI and the system are useful for well-being management?	4	3.5
